# The new normal of web camera theft on campus during COVID-19 and the impact of anti-theft signage

**DOI:** 10.1186/s40163-021-00159-4

**Published:** 2021-10-20

**Authors:** William A. Chernoff

**Affiliations:** Department of Sociology & Criminal Justice, Southeastern Louisiana University, 1205 N. Oak Street, SLU Box 10686, Hammond, LA 70402 USA

**Keywords:** Signage, Theft, Security, Web Camera, Situational Crime Prevention, CPTED, Routine Activities, CRAVED, Classroom, Pandemic

## Abstract

**Objective:**

The opportunity for web camera theft increased globally as institutions of higher education transitioned to remote learning during COVID-19. Given the thousands of cameras currently installed in classrooms, many with little protection, the present study tests the effectiveness of anti-theft signage for preventing camera theft.

**Methods:**

Examined web camera theft at a southern, public university located in the United States of America by randomly assigning N = 104 classrooms to receive either anti-theft signage or no signage. Camera theft was analyzed using Blaker’s exact test.

**Results:**

Classrooms not receiving anti-theft signage (control) were 3.42 times more likely to exhibit web camera theft than classrooms receiving anti-theft signage (medium effect size).

**Conclusions:**

Using classrooms as the unit of analysis presents new opportunities for not only future crime prevention experiments, but also improving campus safety and security. Also, preventing web camera theft on campus is both fiscally and socially responsible, saving money and ensuring inclusivity for remote learners.

## Introduction

The onset of and adaptation to the global coronavirus pandemic required a dramatic reorganization of the routine activities of everyday academic life (Ali, 2020; Toquero, 2020). Where once there were only a few classrooms equipped with remote capabilities, entire campuses were transformed to accommodate online access (Laserfiche, 2020). More web cameras were installed in classrooms during this time than ever before, often on short notice (Skopec, 2020), and with little concern for the possibility of theft.

Clarke (2000) defines a “hot product” as one that is concealable, removable, available, valuable, enjoyable, and disposable—and web cameras possess all these qualities. Web cameras are attractive to thieves because they are lightweight and can fit into a pocket. They are also available all over campus, with many classrooms accessible well into the evening. And they are valuable targets. An entry level web camera can cost around $50, while a top-of-the-line camera can run for $200 or more.

But what made web cameras particularly vulnerable to theft was rooted in the logic of supply and demand. Faculty needed web cameras so they could attend department meetings, hold office hours, and provide remote instruction, if not on campus. And students needed cameras, so they could attend classes and meet with professors. The demand for web cameras soared during this time, and supplies were lacking (Baraniuk, 2020). Anyone looking to make some fast cash could easily sell a web camera on eBay, a major online auction, at more than double its original retail value (Graham, 2020), and it did not matter where the camera came from.

More than just the financial costs associated with theft, web camera theft places a tremendous burden on student learning. Imagine a faculty member entering a classroom, looking to stream a lecture, and the web camera is missing. For students attending remotely, class would be cancelled. Inclusivity is a top priority at any college or university. So, a stolen web camera means that those impacted most by the virus are denied access to their education.

The present study adds to the literature on theft during the pandemic (Felson et al., 2020; Payne et al., 2021) and is of timely importance as more universities transition back to face-to-face instruction. The combination of more people using classrooms means both web cameras and online/hybridized learning are at greater risk. Recognizing these stakes, the present study examined the effect of anti-theft signage across a southern, public university located in the United States. Evidence from a randomized control trial shows that anti-theft signage can have a significant and meaningful impact, providing an effective layer of protection against web camera theft on campus.

## Campus theft prevention

Data from the Federal Bureau of Investigation’s Uniform Crime Reporting Program routinely rates theft as the number one most reported crime on the university campus, comprising over 80% of all known campus offenses in the United States (FBI, 2019). Theft is experienced by students, faculty, and staff and can result in increased fear of crime, decreased academic performance, and, at its most extreme, severe emotional distress (e.g. post-traumatic stress disorder) (Barton et al., 2010). And individuals are not the only ones harmed by theft. The university itself is strained, both financially and in terms of the services provided, when books, academic equipment, and electronic devices are stolen (Edwards, 2018; Ekere et al., 2019; Harrison, 1996; Kaplan, 2021; McGuin, 2010; Miller & Veltri, 2001).

Several studies have sought to understand how the built environment itself affects experiences with theft (Bola, 2020; del Carmen & Stretesky, 1997; Jeffrey, 1971; McGrath et al., 2014; Ouverson, 2016; Tseng et al., 2004). According to del Carmen and Stretesky (1997), locations on campus with low visibility, easy escape, and few barriers to the desired targets are particularly conducive to theft. These environmental conditions were largely supported by Tseng et al. (2004), who showed improving campus parking garage lighting and restricting access reduced reports of theft by over 50%.

While the work of del Carmen and Stretesky (1997) and Tseng et al. (2004) suggest a connection between seclusion and theft, McGrath et al. (2014) observed that most thefts occurred in highly populated areas. Buildings with higher levels of foot traffic accounted for 72.8% of the property crimes reported. That said, greater population density, encouraged by the built environment, could add a layer of anonymity, facilitating easy escape for thieves, an important factor suggested by del Carmen and Stretesky (1997).

Attention to the built environment has also introduced new questions about the meaning students, faculty, and staff attach to the different locations around campus, and how this understanding influences theft (Kijanczuk, 2014; Simmons, 2018). According to Kijanczuk (2014), students know that theft is likely to occur if they leave their belonging unattended in libraries and dining areas (85% and 98%, respectively). However, this vigilance is not uniformly upheld across campus. Fifty-four percent of students viewed classrooms as a safe place, where they believed they could leave their belongings unattended (Kijanczuk, 2014).

Focusing on the situational nature of theft, campus crime scientists have sought to identify theft prevention strategies tailored to different contexts (Chernoff, 2020, 2021; Clarin et al., 2014; Nettle et al., 2012; Wortley & McFarlane, 2011), including libraries (McKay, 2008; Simmons, 2018; Wortley & McFarlane, 2011), parking lots (del Carmen & Stretesky, 1997; Tseng et al., 2004), classrooms (Chernoff, 2020, 2021), bicycle racks (Nettle et al., 2012; Sidebottom et al., 2009), and green spaces (Clarin et al., 2014).

In some cases, greater emphasis is placed on the targets of theft themselves (Chernoff, 2020; 2021; Clarin et al., 2014; Fanno, 1997; Kleberg, 2002; Wortley & McFarlane, 2011; Zhang et al., 2014). Wortley and McFarlane (2011) argued that, in the library context, print cards are necessary for printing assignments, and needed with greater urgency as deadlines approach. They showed that theft could be prevented by labeling print cards with the names of the rightful owner.

In other cases, research has focused on modifying the context itself (Nettle et al., 2012; Wortley & McFarlane, 2011). Bicycle theft is a major problem for many college campuses—and many bicycles are stolen from designated bicycle parking areas (Bola, 2020; Kleberg, 2002; Nettle et al., 2012; Ouverson, 2016). To make this context more adverse to theft, Nettle et al. (2012) employed signage showing watching eyes and the words “Cycle thieves, we are watching you.” Compared to baseline, they observed a significant reduction in theft in the experimental locations.

It is the situational aspect of theft that Clarke (2004, p. 24) sought to highlight when he pointed out that “residential burglars do not generally steal disposable razors blades, but shoplifters do target these.” This axiom means that theft prevention cannot seek easy answers, ones that apply to all targets in all places. But prevention efforts can draw inspiration from other contexts and targets, test different strategies, and discover custom anti-theft solutions across the college campus.

### Theft of campus technology

Most literature on campus technology theft largely focuses on strategies for preventing the theft of laptops and other devices among students, faculty, and/or staff (Bell, 2012; Kijanczuk, 2014; McKay, 2008; Simmons, 2018; Zhang et al., 2014). And while literature exists about preventing the theft of university property (Edwards, 2018; Ekere et al., 2019; Harrison, 1996; Kaplan, 2021; McGuin, 2010; Miller & Veltri, 2001; Zhang et al., 2014), surprisingly little of it focuses on electronic devices.

Perhaps the most thorough discussion on protecting campus technology comes from an article published by Harrison (1996) in the magazine Business Officer. Harrison (1996) argued that the theft of electronic equipment, everything from computers to ink cartridges, can be prevented by using locks and bolts, police patrols, tracking keys/controlling access, alarms, closed circuit television, holding offenders accountable, and marking property. While these strategies are promising, little empirical evidence was provided demonstrating their effectiveness.

Notably, an important challenge with campus technology theft highlighted by Harrison (1996) is the inability to secure all places at all times. According to Chuck Horton, police chief at the University of Georgia, “a thief can find expensive equipment on virtually every floor of every building—much of it small enough to fit into a book bag” (see Harrison, 1996, p. 48). While empirical studies on campus technology theft are sorely lacking, efforts to control the theft of technology on campus should also consider strategies that can exert an effect even when campus police and security are located elsewhere.

## Signage and theft

The Crime Prevention Through Environmental Design (CPTED) literature suggests that the built environment can have a profound impact on theft (Brantingham & Brantingham, 1995; Clarke, 1997; Ekblom, 2000). While it is recommended to include security measures at the design stage (Hughes & Gamman, 2003), oversights can be addressed after the fact. One popular means for doing so is to post anti-theft signage in high-risk areas (Circo & McGarrell, 2020; Hayes et al., 2012, 2019; McKay, 2008; Nettle et al., 2012; Simmons, 2018). Signage can prevent theft because it signals to potential thieves that capable guardians are actively monitoring certain locations, and possibly close at hand (Cohen & Felson, 1979; Cornish & Clarke, 1986).

Cornish and Clarke (2003) also suggested that signage is an effective means for preventing theft, though the reason behind its influence depends on the type of message presented. Theft can be prevented by employing controlling prompts, reminders of the normative behavior expected in certain locations (Cope & Allred, 1991; Cope et al., 1995; Matijosaitiene & Dambriunas, 2015). And signage can encourage compliance by exuding controlling pressures (Cornish & Clarke, 2003). People can be persuaded not to engage in theft if they believe that that crime goes against their values.

While the framework proposed by Cornish and Clarke (2003) tends to view signage as a means for deterring thieves, a major gap in the literature is on what signage can do to encourage guardianship among would-be victims (Johnson et al., 2008; van Lierop et al., 2015). van Lierop et al., (2015, p. 20) argued “racks with prominent signage showing proper locking technique” can encourage potential victims to protect their valuables (though no empirical evidence was provided). But more than just encouraging self-protection, signage can also encourage potential by-standers to take action. While little research exists, Bromley (1997) argued that signage can encourage students, faculty, and staff to be more vigilant against theft and report any suspicious behaviors they observe.

### Statement of hypotheses

The situational crime prevention literature proposes several reasons why signage should prevent theft. The act of hanging signs changes the built environment and suggests to potential thieves the presence of capable guardians, individuals actively investing time and energy into preventing theft in certain locations. But the messages used are also tremendously important. The messages on the signs can encourage normative behavior and/or provide people with opportunities to express their values for security and theft prevention. The present study contends that signage encouraging citizens toward greater security is an effective means for preventing theft—and one sorely in need of further systematic study.

More specifically, the present study contends that anti-theft signage will dissuade theft where it is posted. It is expected that the presence of anti-theft signage in classrooms encouraging security will decrease the likelihood that web cameras will be stolen; and that this effect will be greater than what is observed in classrooms without such signage. In other words, the proportion of web camera thefts prevented will be greater among classrooms with anti-theft signage than among those lacking anti-theft signage.

$$\begin{gathered} {\text{H}}_{0} :{\text{ P}}_{{{\text{Signage}}}} = {\text{ P}}_{{\text{No signage}}} \hfill \\ {\text{H}}_{{1}} :{\text{ P}}_{{{\text{Signage}}}} > {\text{ P}}_{{\text{No signage}}} \hfill \\ \end{gathered}$$

## Method

Southeastern Louisiana University (SELU), a southern, public university located in the United States of America, was chosen out of convenience as the site for the present study. Located in the city of Hammond, Louisiana (affectionately known as “Hammond America”), SELU is a major employer in the area, surpassed only by North Oaks Health System, a large medical center. The university is located near the city’s downtown area and borders mixed residential and commercial areas to the south, east, and west. To the north, the campus exits onto a major city boulevard, providing easy access to the several thoroughfares and highways.

Enrollment-wise, there are 13,490 undergraduate students and 971 graduate students at SELU, and 2,359 students live on campus in residential halls. SELU is primarily a teaching university, boasting a low student to faculty ratio (19:1). Demographically, more students identify as female (64.1%), White non-Hispanic (64.2%), and non-international (98.8%). Due to COVID-19, more classes were available remotely and fewer students were physically on campus, but SELU remained dedicated during this time to providing students with face-to-face instruction. Unless marked for social distancing, desks in classrooms were generally occupied by students during the study period.

Security at SELU is largely provided by University Police Department. Officers regularly patrolling the campus by car and bicycle and are available through a dedicated phone line (2222) as well as several blue call boxes distributed across the university. Campus buildings and classrooms are locked at night, but otherwise the campus and buildings are accessible without the use of swipe cards, making it possible for members of the public to enter the buildings well into the evening.

Classrooms make up the unit of analysis and the departments providing access to these rooms spanned the campus, consisting of: Educational Leadership and Technology, Communication and Media Studies, Music and Performing Arts, Psychology, World Languages and Cultures, Accounting and Finance, Management and Business Administration, Marketing and Supply Chain Management, Teaching and Learning, Kinesiology and Health Studies, School of Nursing, Biological Sciences, Chemistry and Physics, Computer Science, Industrial and Engineering Technology, Sociology and Criminal Justice, History and Political Science, and Mathematics. In total, N = 104 classrooms were included in this study.

It is worth noting that the present study was conducted as part of a larger anti-theft campaign initiated between the author, Southeastern Social Science Research Center (SSSRC), and University Police Department (UPD) at SELU. Permission to post signage in classrooms was also provided by the Dean of Students. And exemption status was approved by the SELU Institutional Review Board.

A randomized experimental design was used to assign classrooms into one of two groups (Kuehl, 2000). Fifty-two classrooms received anti-theft signage, while the remaining 52 classrooms, comprising the control group, did not. By conducting a randomized controlled trial, several important sources of bias were accounted for (Dezember et al., 2020; Farrington & Welsh, 2005; Lum & Yang, 2005), such as the number of desks, number of course sections, number of unique faculty, floor level, or type of classroom (e.g. computer vs traditional); highlighting the present study’s advantage over observational studies (Gomes et al., 2021; Hayes et al., 2012, 2019; Kyvsgaard & Sorensen, 2020).

The study period consisted of the Fall 2020 semester, a 16 week period covering the dates 8/17/2020—12/4/2020. Prior to the start of the semester, each classroom was observed. Treatment classrooms received the anti-theft signage. Signage was placed near lecterns, increasing visibility and awareness (McKay, 2008). Cameras were documented by writing down serial numbers and attaching a small piece of clear tape to the base of the camera, where it would not be seen. After finals week, the classrooms were observed a second time. During this time, signage was removed, and the camera theft was recorded. Any stolen cameras were not replaced during the experimental period.

To avoid the Hawthorne effect, which occurs when the subjects of a study change their behavior precisely because they know they are being studied, both set up and final observation were conducted only when students, faculty, and staff were not in the classrooms (Roethlisberger & Dickson, 1939). Additionally, Reinhart (2015) cautions that the chance of making a Type I error, incorrectly rejecting a true null hypothesis, increases every time the results of an experiment are analyzed. To hold the Type I error rate at alpha = 0.05, only two observations were made: one during the initial setup and another after the exposure period.

### Measurement of variables

The present study examined the effect of anti-theft signage on web camera theft. Classrooms assigned to the treatment group were coded 1, while the remaining classrooms were assigned to the control group and coded 0. The control group did not receive anti-theft signage. The dependent variable consisted of a dichotomous variable, coded 1 if the original camera remained after the exposure period and coded 0 if stolen.

**Fig. 1:**
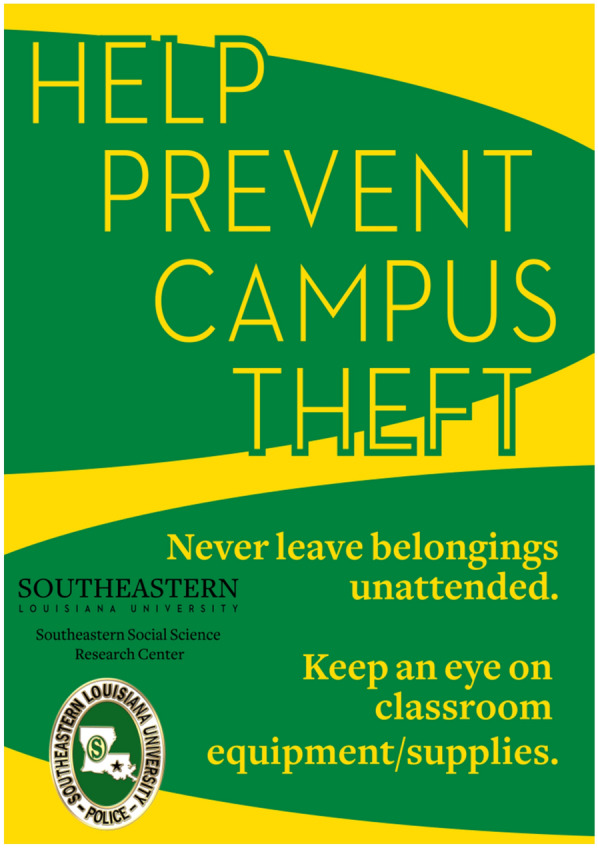
Anti-theft Signage Placed in Treatment Classrooms (A4 page size)

The treatment classrooms in the present study received identical anti-theft signage (see Fig. 1). A substantial amount of scientific work has been conducted on how to design messages, especially in non-criminal contexts such as hazard control (Laughery & Page-Smith, 2006; Lenorovitz et al., 2012; Riley, 2006; Wogalter & Mayhorn, 2008; Wogalter et al., 2002). However, a small but growing body of literature has emerged connecting good message design to the prevention of crime (Prichard et al., 2021). Similar to the criteria compiled by Lenorovitz et al. (2012), the signage used was clear in its anti-theft message (i.e. “Help Prevent Campus Theft), complete in its coverage of theft concerns (i.e. personal and university property), brief its message length, and noticeable in its location (i.e. placed near lecterns). Furthermore, the fonts used on the signage as well as the green and gold color palette were legible and consistent with SELU’s style guide, tailoring the message to the intended audience. Inclusion of the UPD and SSSRC logos added credibility to the source of the message (Wogalter & Mayhorn, 2008).

### Method of data analysis

Blaker’s exact test was used to compare the proportions of web camera thefts prevented for the treatment group (i.e. Signage) to that of the control group (i.e. No signage). This method is appropriate when the design and outcome of an experiment can be represented using a 2 × 2 table (Blaker, 2000). Compared to the more widely used Fisher’s exact test, Blaker (2000) showed his method is preferred because the confidence intervals produced are guaranteed to have the expected level of confidence. Similarly, Fay (2010) showed support for this alternative method, since the test results (i.e. p-value) and confidence intervals calculated using Fisher’s method can contradict one another at times. Blaker’s method (2000) was also preferred because it provides an overall more powerful test, being able to observe an effect with fewer subjects, yielding more accurate confidence intervals.

## Results

Table 1 shows the counts and proportions of web camera theft for the control and treatment classrooms (N = 104). For the 52 treatment classrooms, the ones receiving the anti-theft signage, 47 of the original cameras remained after the exposure period (90.4%). For the control classrooms, the ones not receiving anti-theft signage, fewer cameras were observed. After the exposure period, approximately 73.1% of these cameras (38) remained.

**Table 1:** Web Camera Theft Prevention for Treatment vs Control Classrooms (N = 104)

	Signage	No signage
	Count (Proportion)	Count (Proportion)
Theft	5 (0.096)	14 (0.269)
No theft	47 (0.904)	38 (0.731)

Blaker’s exact test was performed to test whether the effect for the treatment group was statistically significantly greater than that for the control group. Table 2 shows an upper-tailed p-value equal to 0.02. Since the p-value is less than the significance level of alpha = 0.05, sufficient evidence exists to reject the null hypothesis and accept the alternative hypothesis. In other words, there is a statistically significant difference in web camera theft between the classrooms with anti-theft signage and those without—with the likelihood of preventing theft being greater among the treatment classrooms.

**Table 2:** Blaker’s Exact Upper-tailed Test Testing Web Camera Theft Prevention for Treatment vs Control Classrooms (N = 104)

Variable	OR^a^	95% CI	p-value
Signage^b^	3.42	[1.23, ∞]	0.02

OR = odds ratio, CI = confidence interval for odds ratio

^a^ Odds ratio calculated using conditional maximum likelihood estimation

^b^ Reference category is No signage

The conditional maximum likelihood estimated odds ratio equaled 3.42. This statistic suggests that, compared to classrooms receiving no anti-theft signage, classrooms receiving anti-theft signage were 3.42 times more likely to retain their original camera than to have it stolen during the exposure period. In addition to a point estimate, a one-sided 95% confidence interval (1.23, ∞) was constructed to better understand the size of the effect observed. This interval suggests that, with 95% confidence, anti-theft signage was as little as 1.23 times more effective over the long run at preventing web camera theft than doing nothing at all.

## Discussion

The novel corona virus pandemic transformed the educational landscape like never before, moving classes, office hours, and staff meetings online. On short notice, students, faculty, staff, needed web cameras, while at the same time universities worldwide scrambled to purchase and install web cameras in practically every classroom. For some schools, the pandemic was a race to transition to remote learning, and the thought of securing web cameras, an essential component, was often ignored.

The present study examined web camera theft among N = 104 classrooms at a southern, public university located in the United States during a global pandemic. Employing a balanced randomized control treatment design, classrooms were randomly assigned to receive either anti-theft signage or no signage at all (i.e. control condition). Consistent with prior research, results of this experiment showed classrooms without anti-theft signage were significantly more likely to have their web cameras stolen compared to classrooms with this modification (Boba & Santos, 2008; Cialdini, 2005; Cialdini et al., 2006; Davey et al., 2011; Ekblom, 2000; Garrett, 2019; Geller et al., 1983; Nettle et al., 2012; Simmons, 2018; van Lierop et al., 2015; Wortley & McFarlane, 2011).

While understanding risk in conventionally risky areas, such as libraries (McKay, 2008; Simmons, 2018; Wortley & McFarlane, 2011) and parking lots (del Carmen & Stretesky, 1997; Tseng et al., 2004) is important, other locations exist on campus that could generate a false sense of security, encouraging theft (Kijanczuk, 2014). Similar to previous research (Chernoff, 2020, 2021), the present study shows that theft can be reduced in classrooms, areas where risk is generally perceived to be lower (Kijanczuk, 2014). The focus on classrooms is even more important given the recent introduction of lightweight, high demand targets of theft, such as web cameras.

Conceptually, the findings observed are also consistent with the notion of capable guardians from the routine activities approach (Cohen & Felson, 1979). The presence of anti-theft signage, regardless of what is written on it, could ward off potential thieves, since hanging up posters shows that somebody is physically taking action to ensure the safety and security of the campus. Hanging anti-theft signage can, similar to the rational offender approach (Cornish & Clarke, 1986), increase the perceived risk of getting caught and punished, reducing the likelihood of theft.

The words presented on the signage used advised students, faculty, and staff to protect both their own belongings as well as classroom supplies and equipment. Consistent with the situational crime prevention framework, the messages employed could have prevented theft by increasing the perception of the risk of detection (Cornish & Clarke, 1986, 2003). On the one hand, a potential offender might refrain from theft if they believe that the targets worth stealing are actively being monitored (Nettle et al., 2012). For example, Prichard et al. (2021), showed that individuals were less likely to seek potentially exploitive pornography online when shown messages warning that their actions could be observed and traced back to them. On the other hand, the sudden appearance of anti-theft signage itself might give the impression that the person responsible for the signage is actively watching, or within the vicinity, further increasing the likelihood that any theft attempts will be noticed.

That said, future research should examine the difference between direct acts of guardianship (e.g. hanging signage) and indirect acts (e.g. messages claiming guardianship exists). While the present study was unable to separate these effects, evidence could be observed by comparing signage with messages encouraging watchfulness to a more general message. A general message could state “Help Prevent Campus Theft,” and little else. While the present study employed a control condition, studies of theft and signage could further clarify these effects by developing a standardized anti-theft sign, one comparable across studies.

Of the 25 crime prevention techniques proposed by Cornish and Clarke (2003), the effect observed is similar to the notion of providing reminders. Security is a kind of conventional behavior, and it is possible that the messages provided prevented theft by encouraging this behavior. Exerting controlling pressures (Cornish & Clarke, 2003) is also related to the experiment conducted, since the messages used implied that theft was both socially and morally wrong.

More recently, crime scientists have started focusing on the relationship between theft and signage that promotes greater security (Johnson et al., 2008; van Lierop et al., 2015). The language on the signage used departed from the usual use of antagonistic messages (Cialdini, 2003; Nettle et al., 2012), favoring more empowering messages instead. Those reading the signs were provided with actionable tasks they could perform to help prevent theft. They were told to “Never leave belongings unattended” and “Keep an eye on classroom equipment/supplies.”

On the other hand, it is unknown whether the messages conveyed resulted in increased security itself. Future research, however, could investigate securing effects, actions taken by others to prevent theft. In some cases, colleges separate the monitor and peripherals from the central processing unit, locking the computer itself within a podium. While these cabinets are usually equipped with locks, they are not always used. Signage promoting security could produce a securing effect, increasing the likelihood that web cameras, as well as other technology, will be locked away.

Similar to the work of Cialdini et al. (2006), injunctive normative messages were used, commanding those reading the signs to act against theft. But where the authors found that telling people not to steal (i.e. strong focus) had the largest effect, the present study found that the weak focus, telling people how they could prevent theft, to be effective. But this discrepancy is less of a contradiction and more a task for future research. Cialdini et al. (2006) might have observed both effects if they had compared their results to a control group, locations that did not receive anti-theft signage at all.

Another important takeaway from the work of Cialdini et al. (2006) is their focus on interaction effects—the effect of using several anti-theft strategies simultaneously. The present study used two different messages to prevent theft: one instructing people to watch their belongings and the other encouraging people to watch classroom property. However, it is unknown how the two strategies together affected web camera theft. Researchers and practitioners often recommend using multiple anti-theft strategies at a given time (Bell, 2012; Simmons, 2018). But if academics and practitioners want to know when two (or more) strategies counteract each other, or when combining several strategies amplifies the effect, then greater use of experimental design, particularly factorial treatment designs, is sorely needed.

### Limitations

The present study was conducted during extreme circumstances, a global pandemic requiring social distancing and personal protective equipment. One big question then is whether the results observed will generalize. What will happen to web camera theft as restrictions relax? On the one hand, it is uncertain whether academic life will return completely to normal. Advances in remote learning, online/offline hybridization, and web camera theft may be here to stay. On the other hand, these results contain important lessons as universities and colleges not only transition back to in-person instruction, but also manage threats of superbugs in the future.

Similarly, the signage tested could lose its impact over time as students, faculty, and staff grow accustomed to its presence, and stop thinking about its message. Once a habituation threshold has been crossed, it could create “crime harvest” period, a window of opportunity permitting a “mini crime wave” for web camera theft (Clarke, 2004, p. 23; Hughes & Gamman, 2003, p. 38). In fact, it is possible that potential offenders may purposively delay stealing web cameras until the novelty of the signage has worn off. While more research is needed to understand potential habituation effects, agencies are best advised to vary and test new messages to maintain potency.

Displacement effects are also a major concern in any crime prevention effort (Campedelli et al., 2020; Farrell & Birks, 2018; Farrell et al., 2015; Wheeler & Ratcliffe, 2018). Bell (2012) argued that efforts to reduce theft in libraries might cause potential thieves to seek out less protected locations. That said, displacement effects are not guaranteed (Clarke, 2012). As Tseng et al. (2004) observed, improving lighting and reducing access to a parking garage not only decreased theft, but also failed to increase thefts in other parking garages, ones not receiving any anti-theft modifications.

That said, it is equally unknown if there was a diffusion of benefits, where the signage used not only prevented web camera theft, but prevented other thefts as well (Clarke & Weisburd, 1994). Future research could better differentiate displacement effects from diffusion effects by monitoring several desirable targets or outcomes in the classrooms (Sidebottom et al., 2017). Signage could target a specific item, such as dry erase markers (Chernoff, 2020), and see if these thefts increase or diminish in addition to web cameras.

Aesthetics should also be taken into account when creating and hanging anti-theft signage. Garrett (2019) argued that anti-theft signage in art galleries, while effective, can conflict with the goals of the organization, disrupting the visual and emotional feel curated in these spaces. Based on a growing body of evidence on the design of messages (Laughery & Page-Smith, 2006; Lenorovitz et al., 2012; Prichard et al., 2021; Riley, 2006; Wogalter & Mayhorn, 2008; Wogalter et al., 2002), future studies of anti-theft signage should consider the quality as well as the quantity and placement of the messages used—and take pains to ensure they are in line with the goals of the university.

While the effect observed was in the expected direction, actions to reduce theft can have unintended consequences, increasing theft instead of preventing it (Bensley & Wu, 1991; Bushman & Stack, 1996; Weisburd et al., 2011). According to Ekblom (1995), anti-theft signage runs the risk of alerting potential thieves of valuable targets they did not know about. Interviewing pickpockets about anti-theft signage, Ekblom’s (1995) informants reported that people would routinely walk past anti-pickpocket signs and pat down their pockets, showing thieves exactly where people were keeping their valuables. Unlike interventions that inform potential victims and offenders of the target in question (Cialdini et al., 2006; Nettle et al., 2012), the present study sought to diminish potential backfire effects by using a more general anti-theft message—one that did not specify the desirable target.

## Conclusion

In a short period of time, students, faculty, and staff around the world made the transition to remote learning. This unprecedented event dramatically altered the routine activities of campus life and ushered in a new and desirable target of theft: web cameras. The coming semesters promise a return to greater normalcy. But with so much invested in and in place for remote learning, it may truly be a new campus moving forward, equipped for greater remote access, and in need of new anti-theft solutions.

Using a randomized control trial, the present study showed that anti-theft signage can provide an effective layer of protection against web camera theft—and suggesting tremendous cost savings. There are approximately 3,982 institutions of higher education across the United States, each dependent on web cameras (Moody, 2021). Assuming each campus has 100 classrooms, and each classroom is equipped with a $50 web camera, a conservative estimate suggests 3,982 × 100 x $50 = $19,910,000 was spent on web cameras alone. At this rate, even a modest 23% retention in web camera theft (the lower limit of the confidence interval observed), would mean a savings of approximately $4,579,300.

But more importantly, these findings can help ensure the continuity of educational services. Anti-theft signage reduces the likelihood of web camera theft, and in turn, unexpected class cancelations. This means students and faculty can spend more time learning and teaching and less time having to find creative solutions for gaps in the learning process. As campus police and authorities learn to navigate the new normal, relearning what does and does not work, these findings provide a low cost, first step solution for maintaining the security of social life on campus.
